# Supplementary material for: MetaRibo-Seq measures translation in microbiomes
Source: Nat Commun. 2020 Jun 29;11:3268. doi: 10.1038/s41467-020-17081-z (PMC7324362; doi:10.1038/s41467-020-17081-z)
Supplement: Supplementary file 10 — Supplementary Data 7 [file 41467_2020_17081_MOESM10_ESM.zip › File2/Confidence_VeryHigh_Taxonomy/346188_out.krona.html]

Javascript must be enabled to view this page.

members
magnitude
magnitudeUnassigned
count
unassigned
taxon
rank

346188\_out

4

4
2
superkingdom

1239
2
phylum

2
186801
class

order
2
186802

2
541000
family

1263
2
genus


SRS013158\_contig\_number\_900SRS023971\_contig\_number\_contig-100\_999.31033
2
2292374
species

32066
2
phylum

203490
2
class

2
203491
order

family
2
1129771

2
genus
32067

1
species
712357

SRS055378\_contig\_number\_53006

1
species
1785996

SRS149486\_contig\_number\_contig-100\_1370.116131
